# Retrospective audit of antibiotic use in a university general pediatrics department using hospital pharmacy dispensing data

**DOI:** 10.3205/id000075

**Published:** 2021-12-01

**Authors:** Leonie Egle, Katharina Sauter, Svenja Ockfen, Manfred Haber, Sören Becker, Gudrun Wagenpfeil, Michael Zemlin, Sascha Meyer, Arne Simon

**Affiliations:** 1 Pediatric Hematology and Oncology, Children’s Hospital Medical Center, Saarland University Hospital, Homburg/Saar, Germany; 2 Pharmacy, Saarland University Hospital, Homburg/Saar, Germany; 3 Center for Infectious Diseases, Institute of Medical Microbiology and Hygiene, Saarland University Hospital, Homburg/Saar, Germany; 4 Institute for Medical Biometry, Epidemiology and Medical Informatics, University Medical Center, Saarland University, Campus Homburg, Homburg, Germany; 5 Department Clinic for General Pediatrics and Neonatology, Children’s Hospital Medical Center, Saarland University Hospital, Homburg/Saar, Germany

**Keywords:** antibiotic use, pharmacy dispensing data, general pediatrics, neonatal and pediatric intensive care unit (NPICU), antibiotic stewardship

## Abstract

Antibiotics are among the most frequently prescribed drugs in children’s hospitals, which is why regular monitoring of antibiotic use in hospitals is of great importance. This retrospective audit (60 months, January 2014 – December 2018) analyzes the antibiotic consumption at a university inpatient department of general pediatrics including neonatal and pediatric intensive care based on pharmacy dispensing data in units of grams per 100 patient days and in Defined Daily Doses per 100 patient days.

The results provide potential targets for Antibiotic Stewardship interventions. Conversely, this audit elicits methodological limitations of the method of antibiotic surveillance in pediatrics recommended by the Robert Koch Institute, Berlin.

## Introduction

Antibiotics are among the most frequently prescribed drugs in pediatric hospitals [1], [2]. The untargeted use of antibiotics without considering the guidance of national recommendations may impair patient safety [3], foster the selection of multidrug-resistant pathogens [4], [5], [6] and increase the risk of *Clostridioides difficile*-associated infections [7]. For these reasons, the regular surveillance of antibiotic use in pediatric hospitals is of great importance [8], [9], and mandatory under §23 of the German Infection Protection Act [10].

The Robert Koch Institute (RKI, Berlin) has published recommendations how hospitals should accomplish this surveillance [11]. The RKI proposes the recording of antibiotic consumption in antibiotic densities (Defined Daily Doses; DDD/100 patient days). These are calculated from the consumption quantities in grams using the Anatomical Therapeutic Chemical (ATC)/Defined Daily Dose (DDD) classification of the World Health Organization (WHO) [12], [13]. However, the DDD concept refers to normal-weight adults (70 kg) and is therefore unsuitable in pediatrics [8], [14], [15], [16]. In 2014, the German Society for Pediatric Infectious Diseases (DGPI) suggested to document antibiotic consumption in pediatric inpatient care facilities in grams per 100 patient days [17].

The audit presented in this paper, which was conducted at a university hospital for general pediatrics including a neonatal and pediatric intensive care unit (NPICU), evaluates antibiotic consumption over a 5-year period based on pharmacy dispensing data. The main objective was to evaluate the method of surveillance concerning antibiotic consumption as suggested by the RKI and to identify targets for antibiotic stewardship (ABS) interventions.

## Methods

### Setting

This retrospective audit presents the antibiotic consumption at a university children’s hospital based on pharmacy dispensing data. The assessment was carried out for the period from January 1, 2014 to December 31, 2018 for the inpatient department of general pediatrics (46 beds) and the neonatal and pediatric intensive care unit (16 beds).

### Hospital pharmacy dispensing data

**Figure 1:**
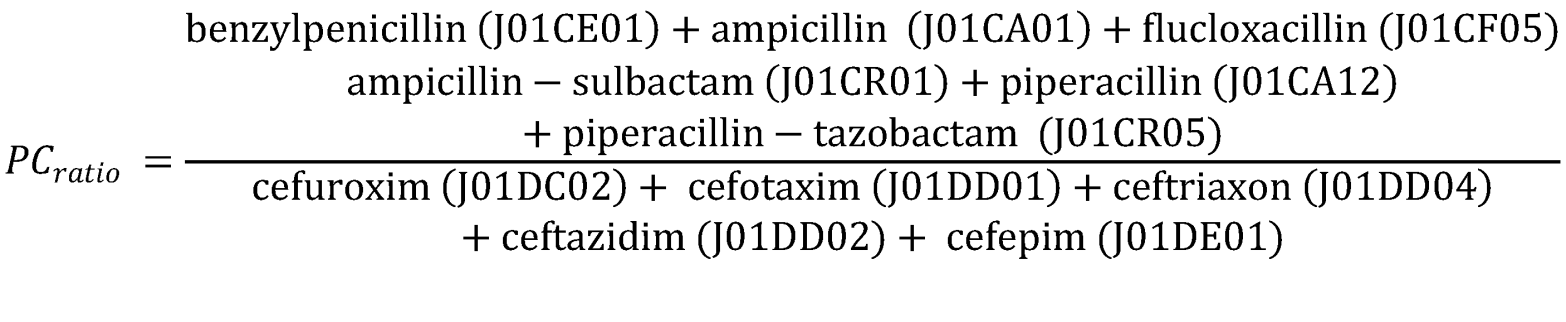
Formula of the penicillin-cephalosporin ratio (PC ratio) calculated referring to pharmacy dispensing data in g/100 inpatient days

The drug consumption data were provided to the hospital pharmacy via the digital evaluation portal PREMAX^®^ AVS from IQVIA™. In this analysis, the “consumption” corresponds to the quantity of the respective antibiotic delivered by the pharmacy to the department. The data includes department-related quarterly consumption data in g and DDD as well as the respective number of stationary patient days. Included in the analysis are the documented consumption data for antibiotics for systemic use (J01) and additionally for the antibiotics rifampicin (J04AB02), oral vancomycin (A07AA09), rifaximin (A07AA11), fidaxomicin (A07AA12) and metronidazole (P01AB01). IQVIA™ calculates the drug consumption data in DDD on the basis of the German official version [18]. Different dosage forms (e.g. intravenous or oral) of baseline data were combined. These values were adjusted to the DDD definition of the WHO ATC System [13]. The DDD of the following five antibiotics could not be calculated from the IQVIA data in accordance with the WHO classification due to different DDD for different administration forms and were therefore excluded from this audit: azithromycin, clarithromycin, clindamycin, erythromycin and cotrimoxazole. A total of 43 antibiotics were included and grouped into 16 antibiotic groups (e.g. broad spectrum penicillins). Defined target parameters of this audit are departmental quarterly and annual antibiotic consumption in grams per 100 patient days (g/100 days) and for correlation in Defined Daily Doses per 100 patient days (DDD/100 days). The annual consumption was calculated from the consumption values of the 4 quarters of a year. Furthermore, the percentage changes of the total consumption as well as the consumption of individual antibiotics between 2014 and 2018 were presented. In addition, a newly introduced antibiotic consumption ratio (the ratio of penicillins to cephalosporins in g/100 days) (Figure 1 (Fig. 1)) was calculated, and piperacillin-tazobactam consumption in g/100 days was correlated with the CMI derived from the diagnosis-related groups (DRG).

### Statistical analysis

Statistical analysis was performed using IBM SPSS Version 25. The evaluation was carried out based on the quarterly consumption values. In addition to descriptive procedures such as the calculation of percentage deviations, linear regression was used as a statistical test to examine the consumption data for significant influences of the variable “year” on antibiotic consumption. A minimum number of 10 cases per influence variable is set as a prerequisite for performing the test. A p-value of less than 0.05 was rated as statistically significant. For the correlation of the units as well as the antibiotic consumption with the CMI, the correlation according to Pearson was performed.

## Results

### Antibiotic consumption

The data derived in this analysis are presented in two tables (Table 1 (Tab. 1), Table 2 (Tab. 2)), as well as here in a descriptive manner. Total cumulative antibiotic consumption showed an annual increasing trend in both departments from 2015 onwards. The minimum consumption in 2015 was 61.3 g/100 days (22.8 DDD/100 days) in the NPICU and in comparison 55.1 g/100 days (23.3 DDD/100 days) in general pediatrics. In 2018, both departments recorded their maximum of 110.1 g/100 days (28.3 DDD/100 days) in the NPICU and 80.0 g/100 days (27.0 DDD/100 days) in general pediatrics.

Except for piperacillin (–46%, both units) and flucloxacillin (–38%, both units) in general pediatrics, an increasing trend in overall penicillin use was observed in both departments from 2014 to 2018. The linear regression showed significant changes over the observation period in general pediatrics with respect to the antibiotic consumption of

- aminopenicillins [amoxicillin, ampicillin: increase per year by 0.45 g/100 days [Confidence interval (CI) 95%: 0.231–0.665; p<0.01) and 0.19 DDD/100 days (CI 95%: 0.048–0.333; p<0.05)], and
- aminopenicillins with beta-lactamase-inhibitor (BLI) in DDD/100 days [intravenous ampicillin-sulbactam, oral sultamicillin: increase per year by 0.17 DDD/100 days (CI 95%: 0.064–0.269; p<0.01)].

However, changes in consumption in g/100 days were not significant (p=0.051).

**Figure 2:**
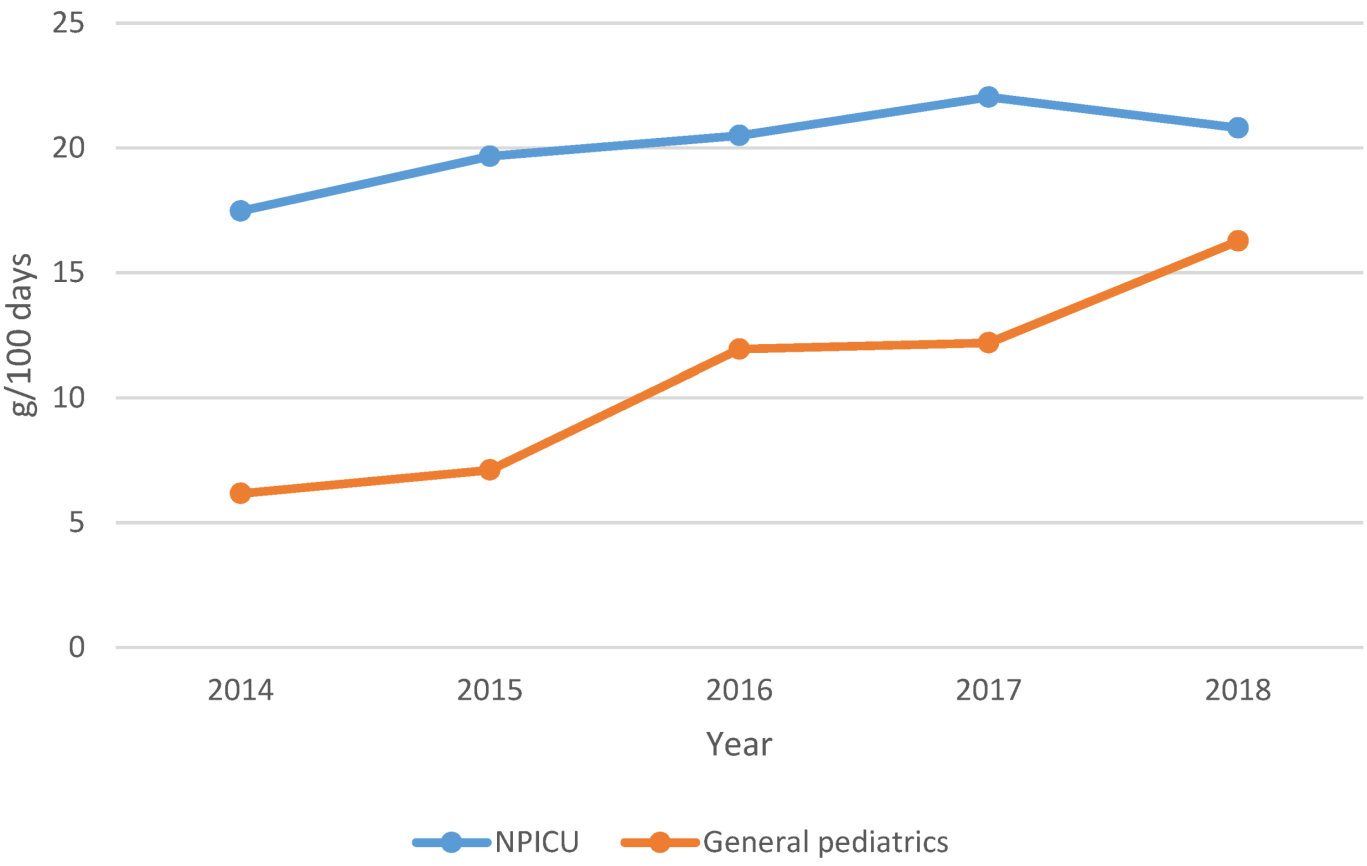
Total consumption of aminopenicillins

In the NPICU, the total consumption of aminopenicillins with BLI (Figure 2 (Fig. 2)) was on average twice as high. Aminopenicillins without BLI were used in the NPICU only in 2017 and 2018. Their share of all aminopenicillins was 3% (g/100 days).

**Figure 3:**
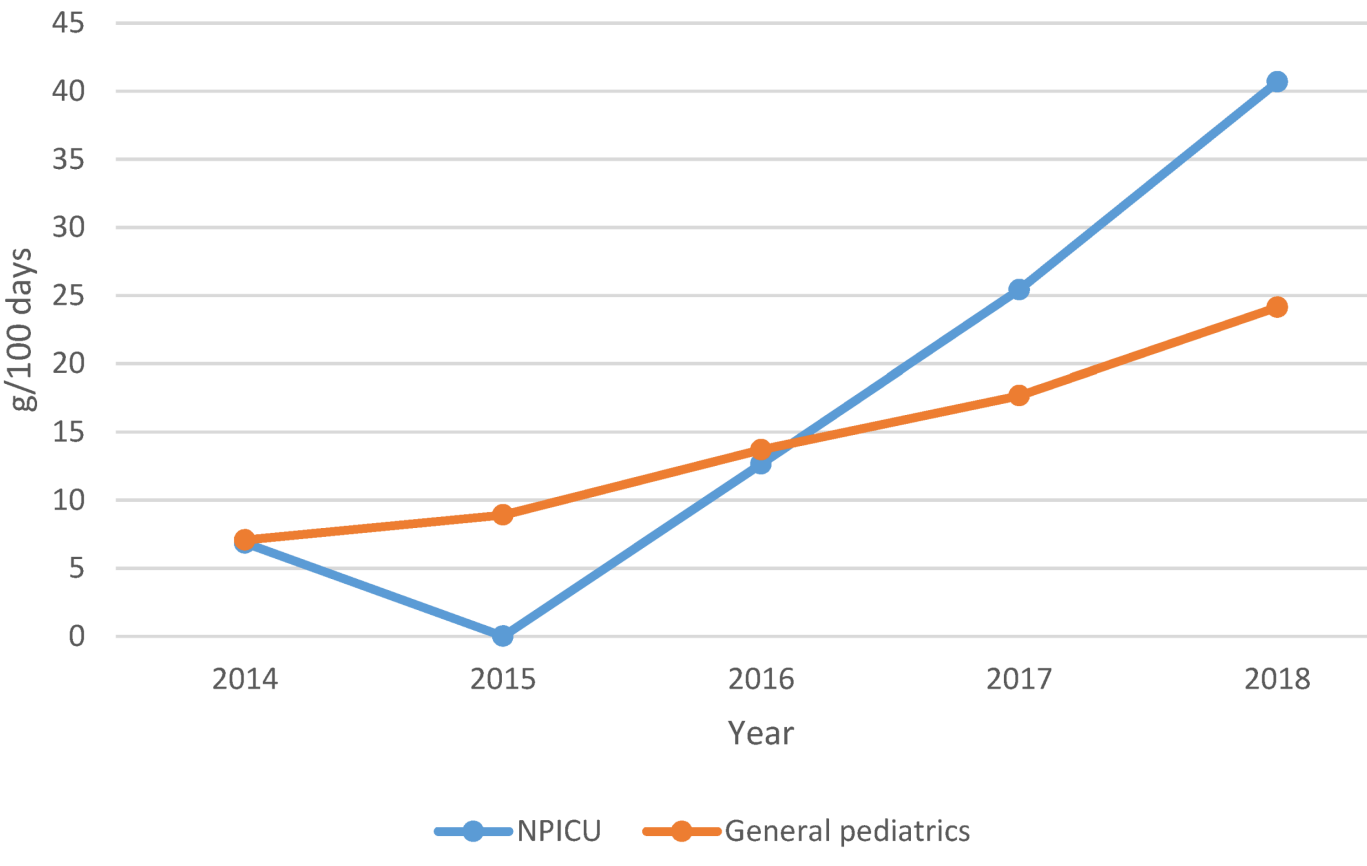
Piperacillin-tazobactam

The increase in piperacillin-tazobactam consumption (Figure 3 (Fig. 3)) was significant in both departments during the observation period. In the NPICU, consumption increased by an average of 8.5 g/100 days per year (CI 95%: 2.640–14.364; p<0.01) or 0.61 DDD/100 days (CI 95%: 0.189–1.027; p<0.01), whereas in general pediatrics consumption increased by 4.31 g/100 days (CI 95%: 2.339–6.171; p<0.01) or 0.31 DDD/100 days (CI 95%: 0.175–0.441; p<0.01).

Both departments showed a decreasing trend in total cephalosporin consumption between 2014 and 2018 (8% to 14%). Group I cephalosporins (cefazolin, cefaclor) were no longer used in the NPICU from 2015 onwards. However, between 2014 and 2018 there was an increasing trend in the consumption of cefuroxime (group II cephalosporine) by 22% (g/100 days) and 21% (DDD/100 days) respectively (p>0.05).

The proportion of cefuroxime in all cephalosporins showed an upward trend from 40% (2015) to 70% (2017 and 2018). Group III cephalosporins (cefotaxime, ceftazidime, ceftriaxone) were used less frequently during the course of the study [decreasing trend by 57% (g/100 days) and 58% (DDD/100 days); p>0.05].

**Figure 4:**
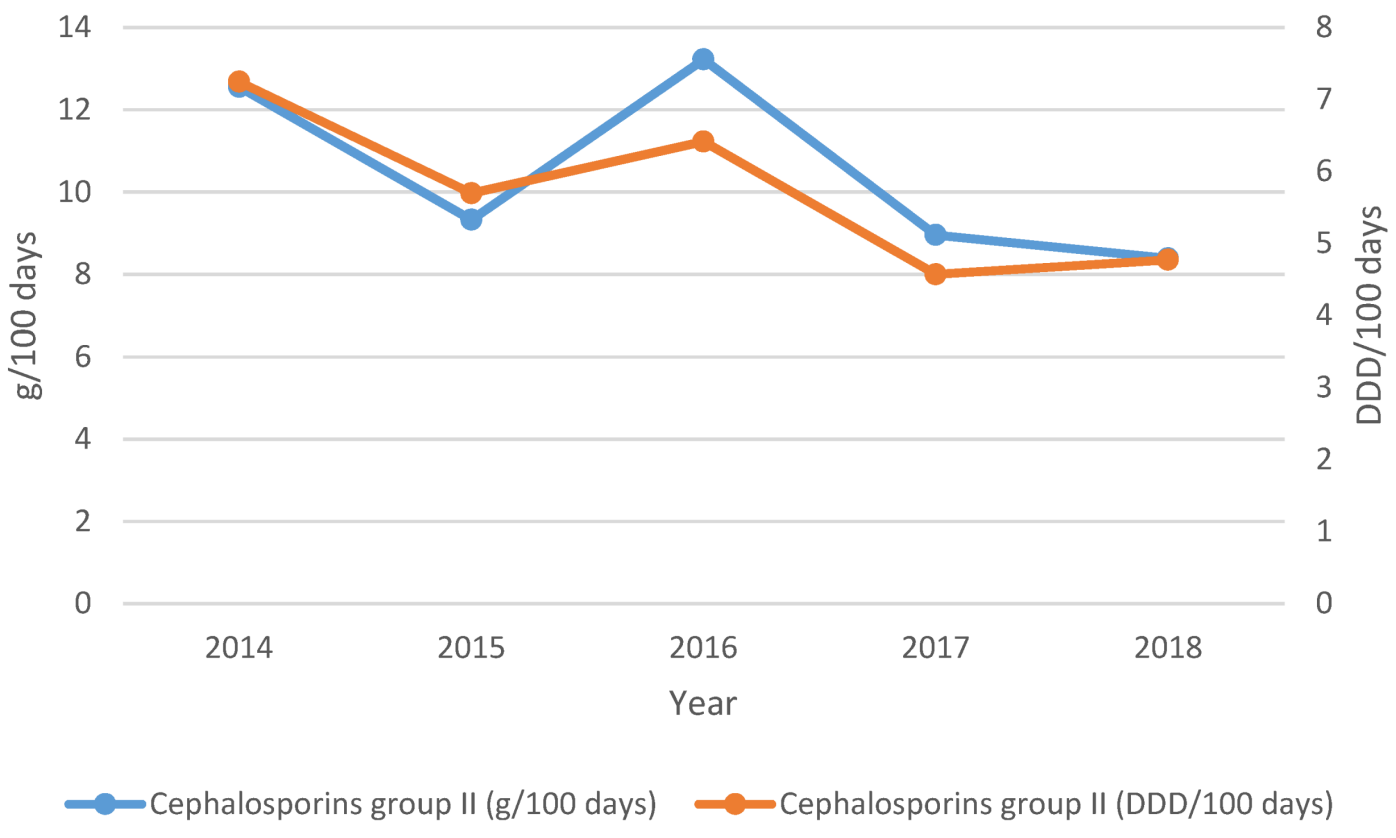
Cephalosporins group II, general pediatrics

In contrast, an increasing trend of 60% (both units) was observed for group I cephalosporins in general pediatrics. The consumption of cefuroxime (group II) (Figure 4 (Fig. 4)) decreased significantly by 0.60 DDD/100 days per year (CI 95%: –1.068 to –0.141; p<0.05) between 2014 and 2018. Changes in consumption in g/100 days were not statistically significant (p>0.05). The relative share of cefuroxime in total cephalosporin consumption in general pediatrics showed a downward trend from 45% (2014) to 33% (2018) over the 4-year observation period. Group III cephalosporins (cefotaxime, ceftazidime, ceftriaxone, cefpodoxime) and group IV cephalosporins (cefepim) showed an insignificant increase of 8% (g/100 days) and 10% (DDD/100 days), respectively, between 2014 and 2018.

The consumption values for carbapenems (meropenem, imipenem-cilastatin) showed a median consumption of 11.3 g/100 days (3.8 DDD/100 days) in the NPICU with only marginal variation over the years. Conversely, in general pediatrics, consumption ranged from 2.1 g/100 days (0.7 DDD/100 days) to 5.0 g/100 days (1.7 DDD/100 days), and recorded an upward trend by 36% (g/100 days) and 33% (DDD/100 days) between 2014 and 2018. The relative share of meropenem with regard to total consumption of carbapenems (g/100 days) was between 93% and 100% in both departments.

A slight downward trend of 26% g/100 days (19% DDD/100 days) in aminoglycoside (tobramycin, gentamicin, amikacin) consumption levels in the NPICU was recognized between 2014 and 2018.

In general pediatrics, the consumption values per 100 days were, on average, twice as high.

The consumption of fluoroquinolones (cipro-, levo-, moxifloxacin; ciprofloxacin generally accounted for the largest proportion) showed a declining trend of 17–27% from 2014 to 2018 in both departments in relation to the unit g/100 days.

In the NPICU, the consumption values of glycopeptides (vancomycin, teicoplanin) were between 2.3 and 4.0 g/100 days (3.1–4.0 DDD/100 days) and showed a slightly increasing trend between 2014 and 2018 (49% g/100 days and 10% DDD/100 days).

**Figure 5:**
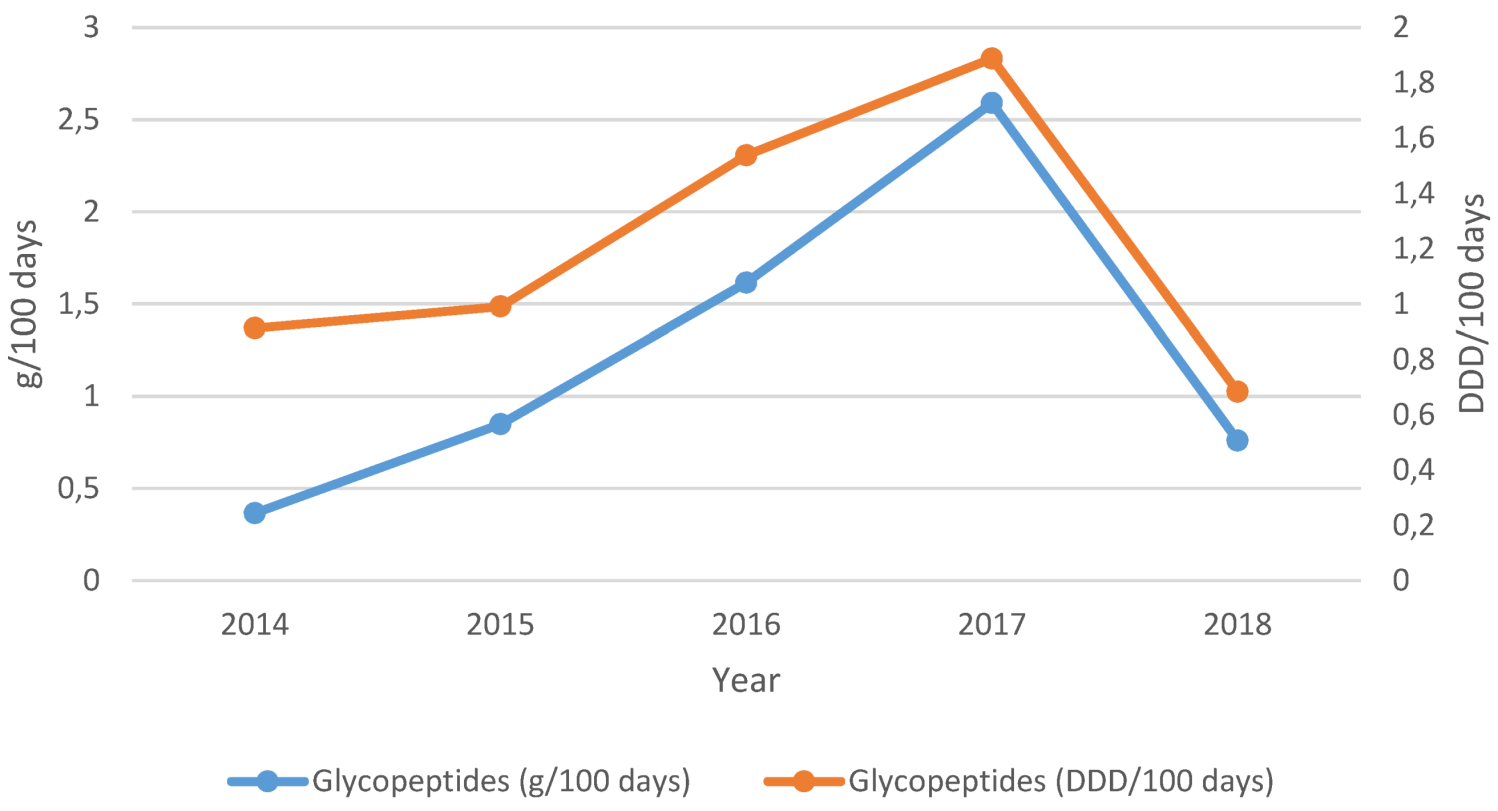
Glycopeptided, general pediatrics

In general pediatrics, consumption increased to a maximum of 2.6 g/100 days (1.9 DDD/100 days) by 2017 and decreased by 71% (g/100 days) and 64% (DDD/100 days) in the following year (Figure 5 (Fig. 5)).

The share of vancomycin in total glycopeptide consumption ranged from 0% (2014) to 89% (2017) in general pediatrics and from 51–76% in the NPICU. As DDD for vancomycin (DDD=2 g) and teicoplanin (DDD=0.4 g) differ substantially and the two antibiotics were consumed in different proportions each year (e.g. 0% vancomycin in 2014 but 80% vancomycin in 2018), the values in g/100 days and DDD/100 days differ without depicting clear longitudinal tendencies.

In general pediatrics, glycopeptide consumption thus increased by 108% (g/100 days) and decreased by 25% (DDD/100 days), without statistical significance, between 2014 and 2018. In the NPICU, an upward trend of 49% (g/100 days) and 10% (DDD/100 days) was observed during the 5-year observation period.

### Penicillin-cephalosporin ratio

**Figure 6:**
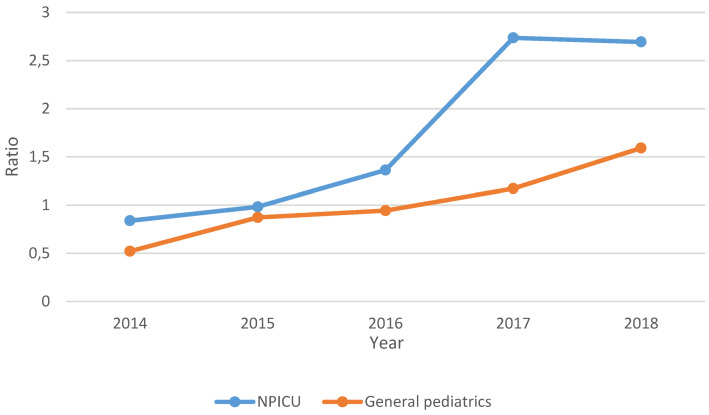
Penicillin-cephalosporin ratio

In both departments, the penicillin-cephalosporin ratio (Figure 1 (Fig. 1)) showed an increasing trend between 2014 and 2018. In the NPICU, the values ranged from 0.8 (2014) to 2.7 (2018), exceeding 1 from 2015 onwards. In general pediatrics, there was an upward trend in the ratio from 0.5 (2014) to 1.6 (2018), reaching values >1 from 2017 onwards (Figure 6 (Fig. 6)).

### Correlation of g/100 days and DDD/100 days 

The correlation of the consumption values in g/100 days and the consumption values in DDD/100 days within different antibiotic groups varied substantially. In both departments, the weakest correlation was found in the glycopeptide group (NPICU: r=0.399, p<0.05; general pediatrics: r=0.693, p<0.01). Strong correlations were found for the carbapenems (NPICU and general pediatrics: r=0.999; p<0.01). The strength of the correlation depends on the number, frequency and quantity of the different antibiotics used in a group, as well as on the differences in DDD factors. The strong correlation in carbapenems can be explained by the very high proportion of meropenem in total carbapenem consumption (93–100%).

## Discussion

In Germany, the Robert Koch Institute is responsible for recommending details concerning the surveillance of antibiotic consumption in hospitals according to §23 (Abs. 4) of the Infection Prevention Act. It advises hospital pharmacies and clinicians affiliated at pediatric inpatient facilities to analyze antibiotic consumption in DDD/100 inpatient days [8], although there is no consensus definition available for DDDs in pediatric patients. In 2013, the German Society for Pediatric Infectious Diseases (DGPI) suggested to overcome this obstacle by using g/100 inpatient days as alternative metric [17].

To our knowledge, this is the first comparative presentation of antibiotic consumption in DDD and in g/100 days in a university-affiliated general pediatric hospital including data from a neonatal and pediatric intensive care unit (NPICU). A point prevalence study by Gharbi et al. in 61 pediatric departments in the UK showed comparable consumption values in DDD/100 days, broken down by different age categories [6]. Buccellato et al. conducted a departmental analysis of pharmacy dispensing data (2004–2011) at 16 hospitals in Italy. Compared to this audit, however, the consumption figures for general pediatrics (in DDD/100 patient days) were more than twice as high (64–77 DDD/100 days) [19].

The regular analysis of antibiotic consumption data in inpatient departments aims at the identification of targets for antimicrobial stewardship [20]. This audit elicits an increase in the consumption of penicillins with a comparatively narrow spectrum of activity (significant increase of aminopenicillins in general pediatrics), which is desirable from an ABS perspective. Conversely, it documents a significant increase in the consumption of the broad-spectrum penicillin piperacillin-tazobactam in both departments, which must be critically examined.

Piperacillin-tazobactam should be used in a specific and guideline-compliant manner for severe infections in order to reduce the selection pressure on extended spectrum ß-lactamase (ESBL)-producing bacteria as executed by extended spectrum cephalosporins [21], [22], [23]. Compared to the group III and IV cephalosporins, piperacillin-tazobactam is often considered a well-suited alternative (e.g. in severe intra-abdominal infections where ceftriaxone and cefotaxime must be combined with metronidazole or in complicated pneumonias in high-risk patients). One definite exception is meningitis, where cefotaxime and ceftriaxone are more suitable due to their better ability to cross the blood-brain barrier.

Any increase in penicillin-based treatments should also be evaluated in conjunction with cephalosporin consumption. In principle, a shift in consumption data from the more broadly effective cephalosporins (groups II, III and IV) to the penicillins is desirable from an ABS perspective [24], [25], [26]. Looking at the consumption values of the cephalosporins, opposing developments of the two departments become clear. In general pediatrics, a decrease of cefuroxime (group II) (significant for DDD/100 days) in combination with an increase in the consumption of group I cephalosporins has to be mentioned. Reasons for this may include increased use of aminopenicillins for respiratory tract infections and group I cephalosporins for perioperative antibiotic prophylaxis.

In the intensive care units, hardly any group I cephalosporins were used, and the consumption of cephalosporins II showed an increasing trend by 22% (g/100 days) and 21% (DDD/100 days) between 2014 and 2018. We are not able to explain this trend without a detailed analysis of the prescription habits in these units.

This development is an important target for ABS while the decrease of broad-spectrum cephalosporins group III and IV (57% g/100 days and 58% DDD/100 days) between 2014 and 2018 demonstrated a positive trend in this unit. The untargeted and prolonged use of group III cephalosporins is associated with an increase in the incidence of *Clostridioides difficile* infections and the selection of resistant pathogens (including Methicillin-resistant *Staphylococcus* *aureus* (MRSA), Vancomycin-resistant enterococci (VRE) and multidrug-resistant Gram-negative pathogens (MRGN) [24], [27], [28], [29], [30]. The results of the penicillin-cephalosporin ratio, which was first introduced in this audit, reflect the increasing trend of penicillin consumption in combination with the overall decreasing use of cephalosporines. This ratio can be programmed in the algorithms used by the hospital pharmacy for the generation of antibiotic consumption reports and can then be easily calculated on demand. A study by Kreitmeyr et al. at four general pediatric wards was even able to reduce the consumption (in days of therapy, DOT/1,000 days) of cephalosporins by 35.5% by an ABS intervention, while the consumption of penicillins could be increased by 15% [24]. In another study, the investigators achieved a reduction of the total consumption and especially of the broad-spectrum antibiotics in DOT/1,000 days in a pediatric and neonatal intensive care unit through ABS interventions [25].

This audit identifies meropenem as the most frequently used carbapenem in both departments (93–100%). Carbapenem consumption is higher in intensive care units, where a higher proportion of patients with complicated, potentially life-threatening or hospital-acquired infections are treated. Nevertheless, due to its frequent empirical use [6] and the risk of selecting carbapenem-resistant enterobacteriaceae (CRE) [31], [32], consumption should be the subject of case-specific analyses in order to assess the indication and duration of treatment. Considering the rising trend between 2014 and 2018 (+36% g/100 days and +33% DDD/100 days), this is particularly true for general pediatrics. Again, the analysis solely referring to pharmacy dispensing data (as recommended by the RKI) does not allow any tentative conclusion why this upward trend occurred in general pediatrics.

The consumption data of the aminoglycosides of the NPICU demonstrated an overall decreasing trend during the observation period (26% g/100 days and 19% DDD/100 days). In contrast, aminoglycoside consumption rates per 100 days are higher in general pediatrics. These differences may be explained by the fact that aminoglycosides in intensive care units are mainly used as empirical standard therapy for early-onset sepsis (ampicillin-sulbactam plus gentamicin). Here, a considerable number of premature and newborn infants with many patient days generate cumulatively only small amounts of consumption due to their low body weight. The decreasing trend in the consumption of fluoroquinolones in both departments can be seen as positive. In pediatrics, fluoroquinolones are mainly suitable for the specific therapy of infections caused by pathogens for which there are no alternatives. The only exception is *Pseudomonas* eradication in cystic fibrosis [33]. Our analysis suggests that in hospitals, where vancomycin and teicoplanin are used, the corresponding glycopeptide consumption should be analyzed separately (Vancomycin vs. Teicoplanin). In our hospital, there is no definite internal standard available for determining which of both glycopeptides should be used preferably. This should be addressed by the ABS team in future initiatives.

When comparing the different metrics, it should be noted that the pharmacy dispensing data do not correspond to the actual patient and case-related antibiotic consumption, neither in g/100 days nor in DDD/100 days. Due to different dosages of antibiotics in pediatrics compared to adults, a large portion of standard preparations (vials for adults) are discarded. The quantities of antibiotics (in g) dispensed by the hospital pharmacy to pediatric wards therefore do not correspond to the doses actually administered [17].

This is a clear limitation concerning the informative value of the RKI recommendation on antibiotic surveillance in pediatric inpatients facilities [8]. The unit g/100 inpatient days (proposed by the DGPI [17]) appears to be more suitable for grouping, comparing and interpreting antibiotic consumption in pediatrics, as it does not depend on an additional conversion factor derived from adult patients (DDD). All these obstacles fade as soon as an electronic data file becomes available for the clinical management of the patients, which improves the documentation of any medication as administered (real patient derived data).

## Conclusions

This audit used pharmacy dispensing data to map a consumption overview of various antibiotics in the general pediatrics department and the NPICU over a period of 5 years. As starting points for future ABS programs, the reduction of the consumption of group II cephalosporins and piperacillin-tazobactam as well as the increase of the use of aminopenicillins without BLI and group I cephalosporins was identified for the NPICU. In general pediatrics, the consumption of group III and IV cephalosporins, piperacillin-tazobactam and carbapenems should be reduced. The audit confirms the existing difficulties in recording antibiotic consumption in pediatrics and considers the use of the unit gram per 100 patient days to be more appropriate for future evaluations. Due to the limitations of pharmacy data, an early introduction of electronic patient documentation systems is essential for providing rapid access to the actual anti-infective quantities administered in grams. The quality of antibiotic prescription should be investigated with repeated point-prevalence surveys [6], [34].

## Notes

### Ethical approval

No patient-specific data were stored in the departmental order process of the pharmacy, and therefore neither in the data set of PREMAX^®^ AVS from IQVIA. The Ethics Committee of the Medical Association of Saarland (Saarbrucken, Germany) confirmed that no ethics approval was required for this audit.

### Authors’ contributions

AS, MH and SB developed the concept of this audit, LE collected and analyzed the data, GW performed the statistical analysis. All authors contributed to the drafting and the finalization of the manuscript.

### Competing interests

The authors declare that they have no competing interests.

## Figures and Tables

**Table 1:**
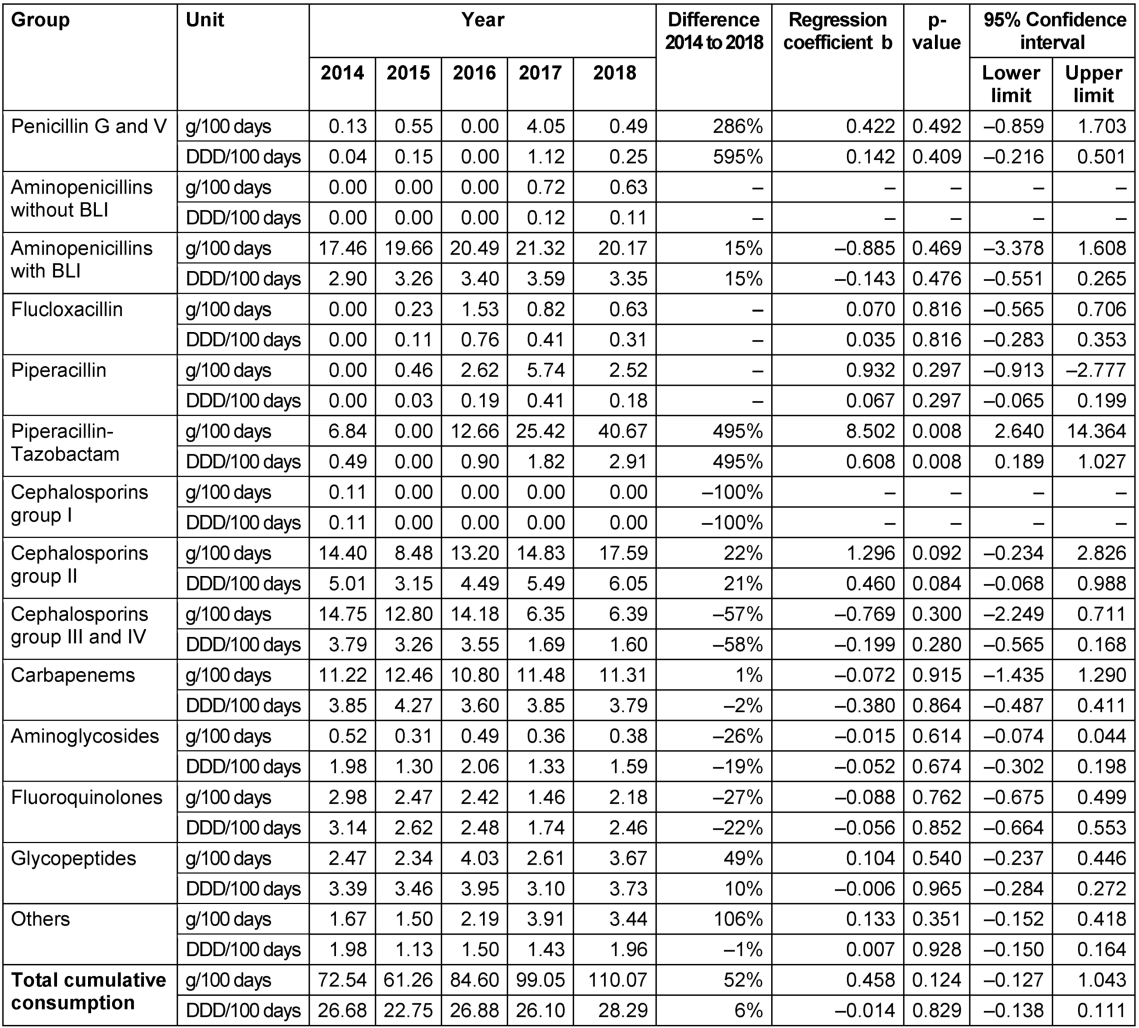
Antibiotic consumption derived from pharmacy dispensing data in the neonatal and pediatric intensive care units (NPICU) 2014–2018

**Table 2:**
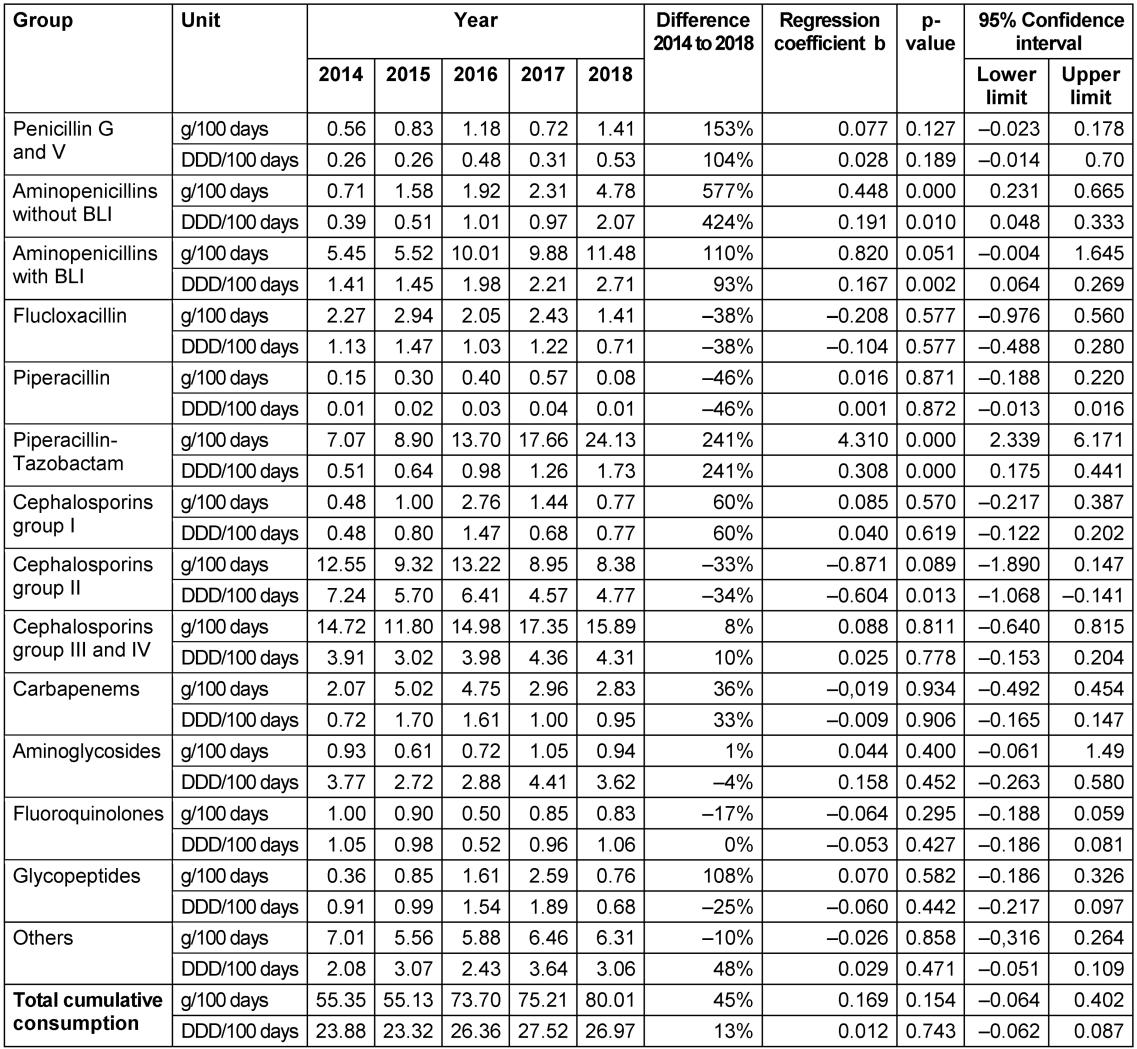
Antibiotic consumption derived from pharmacy dispensing data in the general pediatric wards 2014–2018
